# Lung distribution of gas and blood volume in critically ill COVID-19 patients: a quantitative dual-energy computed tomography study

**DOI:** 10.1186/s13054-021-03610-9

**Published:** 2021-06-21

**Authors:** Lorenzo Ball, Chiara Robba, Jacob Herrmann, Sarah E. Gerard, Yi Xin, Maura Mandelli, Denise Battaglini, Iole Brunetti, Giuseppe Minetti, Sara Seitun, Giulio Bovio, Antonio Vena, Daniele Roberto Giacobbe, Matteo Bassetti, Patricia R. M. Rocco, Maurizio Cereda, Rahim R. Rizi, Lucio Castellan, Nicolò Patroniti, Paolo Pelosi, Collaborators of the GECOVID Group, Mattia Bixio, Angelo Gratarola, Paolo Frisoni, Maurizio Loconte, Alexandre Molin, Giulia Orefice, Elena Ciaravolo, Federico Costantino, Dario Battioni, Gerolama Buconte, Alessandro Casaleggio, Giuseppe Cittadini, Luca Dogliotti, Veronica Giasotto, Sara Perissi, Maria Pigati, Elena Santacroce, Federico Zaottini, Chiara Dentone, Lucia Taramasso, Laura Magnasco, Matilde Bastianello

**Affiliations:** 1 Department of Surgical Sciences and Integrated Diagnostics (DISC), University of Genoa, Viale Benedetto XV 16, Genoa, Italy; 2 Anesthesia and Intensive Care, Ospedale Policlinico San Martino, IRCCS per l’Oncologia e le Neuroscienze, Genoa, Italy; 3 Department of Biomedical Engineering, Boston University, Boston, MA USA; 4 Department of Radiology, University of Iowa, Iowa City, IA USA; 5 Department of Radiology, Perelman School of Medicine, University of Pennsylvania, Philadelphia, PA USA; 6 Oncology and Interventional Radiology Unit, Ospedale Policlinico San Martino, IRCCS per l’Oncologia e le Neuroscienze, Genoa, Italy; 7 Infectious Diseases Unit, Ospedale Policlinico San Martino, IRCCS per l’Oncologia e le Neuroscienze, Genoa, Italy; 8 Department of Health Sciences (DISSAL), University of Genoa, Genoa, Italy; 9 Laboratory of Pulmonary Investigation, Carlos Chagas Filho Institute of Biophysics, Federal University of Rio de Janeiro, Rio de Janeiro, Brazil; 10 Department of Anesthesiology and Critical Care, Perelman School of Medicine, University of Pennsylvania, Philadelphia, PA USA; 11 Radiology Department, Ospedale Policlinico San Martino, IRCCS per l’Oncologia e le Neuroscienze, Genoa, Italy

**Keywords:** COVID-19, Dual energy computed tomography, ARDS, Lung imaging

## Abstract

**Background:**

Critically ill COVID-19 patients have pathophysiological lung features characterized by perfusion abnormalities. However, to date no study has evaluated whether the changes in the distribution of pulmonary gas and blood volume are associated with the severity of gas-exchange impairment and the type of respiratory support (non-invasive versus invasive) in patients with severe COVID-19 pneumonia.

**Methods:**

This was a single-center, retrospective cohort study conducted in a tertiary care hospital in Northern Italy during the first pandemic wave. Pulmonary gas and blood distribution was assessed using a technique for quantitative analysis of dual-energy computed tomography. Lung aeration loss (reflected by percentage of normally aerated lung tissue) and the extent of gas:blood volume mismatch (percentage of non-aerated, perfused lung tissue—shunt; aerated, non-perfused dead space; and non-aerated/non-perfused regions) were evaluated in critically ill COVID-19 patients with different clinical severity as reflected by the need for non-invasive or invasive respiratory support.

**Results:**

Thirty-five patients admitted to the intensive care unit between February 29th and May 30th, 2020 were included. Patients requiring invasive versus non-invasive mechanical ventilation had both a lower percentage of normally aerated lung tissue (median [interquartile range] 33% [24–49%] vs. 63% [44–68%], *p* < 0.001); and a larger extent of gas:blood volume mismatch (43% [30–49%] vs. 25% [14–28%], *p* = 0.001), due to higher shunt (23% [15–32%] vs. 5% [2–16%], *p* = 0.001) and non-aerated/non perfused regions (5% [3–10%] vs. 1% [0–2%], *p* = 0.001). The PaO_2_/FiO_2_ ratio correlated positively with normally aerated tissue (*ρ* = 0.730, *p* < 0.001) and negatively with the extent of gas-blood volume mismatch (*ρ* = − 0.633, *p* < 0.001).

**Conclusions:**

In critically ill patients with severe COVID-19 pneumonia, the need for invasive mechanical ventilation and oxygenation impairment were associated with loss of aeration and the extent of gas:blood volume mismatch.

**Graphic abstract:**

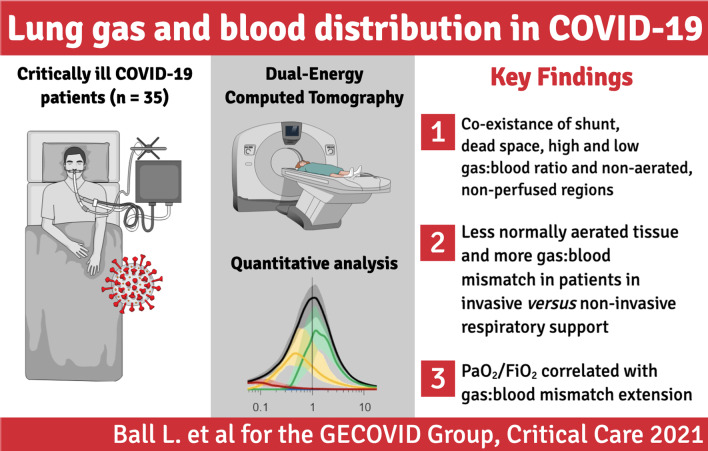

**Supplementary Information:**

The online version contains supplementary material available at 10.1186/s13054-021-03610-9.

## Introduction

In December 2019, a disease (COVID-19) caused by a novel coronavirus (SARS-CoV-2) emerged in China; it has since spread globally, causing a pandemic. COVID-19 patients present with a variety of clinical manifestations, including severe hypoxemic respiratory failure requiring mechanical ventilation [1–4]. Although these patients typically meet criteria for the acute respiratory distress syndrome (ARDS), peculiar pathophysiological features have been identified [5], which require specific therapeutic strategies [6, 7]. COVID-19 patients often display severe hypoxemia due to high shunt fraction over a wide range of lung compliance, and chest computed tomography (CT) findings do not always fully explain the degree of gas-exchange impairment [8, 9]. There have been contrasting reports concerning differences between the pathophysiology of COVID-19-related ARDS and ARDS due to other causes [10, 11], with relevant implications for mechanical ventilation settings [12].

The radiological hallmarks of COVID-19 are ground-glass opacities, often overlapping with areas of lung consolidation [13]. It has been hypothesized that ground-glass areas represent high perfusion, which results in elevated shunting [6, 8, 14]. Large ventilated, non-perfused areas have also been reported [15], even in the absence of pulmonary embolism [16]. This suggests that shunt, areas with both increased and decreased ventilation-perfusion ratio, and pulmonary microthrombi might coexist [17]. Contrast-enhanced, dual-energy computed tomography (DECT) is an imaging technique capable of producing quantitative iodine density maps and depicting regional pulmonary blood distribution, and has been proposed as a tool to measure both lung aeration and perfusion in pulmonary diseases [18] including COVID-19 [16].

The association of changes in lung aeration and perfusion with gas-exchange impairment and disease severity in COVID-19 requires investigation. Within this context, we conducted a retrospective cohort study with the aim of quantifying lung aeration and perfusion changes in critically ill patients with severe COVID-19 pneumonia. We hypothesized that the severity of COVID-19 pneumonia, as reflected by the requested respiratory assistance (non-invasive *versus* invasive respiratory support) and the degree of gas-exchange impairment, was associated with the extent of gas:blood volume mismatch.

## Methods

This retrospective cohort study was conducted in a university-affiliated hospital in Genoa, northern Italy during the peak phase of the first COVID-19 pandemic wave. The study protocol was approved by the ethics review board (Comitato Etico Regione Liguria, protocol n. 163/2020) and the need for written informed consent was waived for retrospective data. The study is reported in accordance with the *STrengthening the Reporting of OBservational studies in Epidemiology* (STROBE) and *REporting of studies Conducted using Observational Routinely-collected health Data* (RECORD) [19] recommendations.

### Patient inclusion flow and data collection

This study included consecutive critically ill COVID-19 patients, as confirmed by SARS-CoV-2 polymerase chain reaction on nasopharyngeal swab specimens, admitted from February 29th to May 30th, 2020, who underwent a DECT scan during their ICU stay. Indications for DECT were the need to guide mechanical ventilation and anticoagulation strategy in presence of worsening of gas exchange or suspicion of pulmonary embolism. Exclusion criteria were acquisition of DECT after May 30th, 2020, and the presence of bone artefacts due to the acquisition of DECT scan with arms down. Demographic, epidemiological and clinical data were collected from electronic medical records, both at the time of ICU admission and on the day of DECT. Data on chronic therapies and comorbidities were retrieved from the clinical records. Patients were grouped according to their severity as reflected by the level of respiratory support received on the day of the DECT scan (non-invasive respiratory support versus invasive mechanical ventilation) and their survival monitored until ICU discharge. The choice of delivering invasive mechanical ventilation was based on the evaluation of clinical parameters including severity of hypoxemia and dyspnea, failure of non-invasive respiratory support and absence of contraindications to ICU admission. The study period was characterized by extremely high healthcare resources use and non-invasive ventilation was also used as a bridge to intubation and ICU admission in severe patients in case of ICU beds shortage. Moreover, high-flow oxygen therapy was not yet extensively implemented. In this context, DECT was introduced in COVID-19 patients at our institution as a standardized clinical protocol to optimize therapeutic management. The Additional file 1 reports further details on the clinical context, indications and rationale for the use of DECT in minute detail. We computed the ventilatory ratio as minute ventilation (ml/min) × PaCO_2_/(predicted body weight × 100 × 37.5 mmHg) [20].

### Protocol for DECT analysis

Pulmonary parenchyma and vessel segmentations were obtained using multi-resolution convolutional neural networks excluding blood vessels larger than 1 mm [21], followed by manual refinement as necessary. Since our segmentation method excluded blood vessels, the absolute values of lung volumes and weight might not be directly comparable to those obtained in conventional CT studies, where blood vessels are frequently misclassified as non-aerated tissue [22, 23]. Spatial distributions of gas and blood within the lung mask were assessed by virtual non-contrast (VNC) and pulmonary blood volume (PBV) images [24], computed via a three-material decomposition algorithm [25], as detailed in the online supplement. Briefly, DECT simultaneously acquires two scans with different radiation energies that interact differently with iodinated contrast medium, and the two scans are analyzed to reconstruct the spatial distribution of iodine within the lungs. Three regions of interest (ROIs) of equal lung tissue weight [22, 26] were partitioned by two planar cuts along either the ventral-dorsal or craniocaudal axis. Lung volume, pulmonary gas volume (Vgas), and aeration analyses were computed based on the VNC image, dividing lung compartments into hyper-, normally, poorly, and non-aerated, according to attenuation thresholds commonly used in ARDS studies [22]. The presence of macroscopic pulmonary embolism was evaluated by two radiologists (GM and SS).

### Lung gas:blood volume matching analysis

We used VNC and PBV maps to divide the lung into compartments with homogeneous characteristics of gas and blood distribution. We defined as *non-perfused* tissue the percent of lung mass composed by voxels in which the PBV was below the limit of detection of the DECT technique (PBV < 1 HU), thus identifying regions of the lung not reached by the contrast medium. Lung gas:blood volume mismatch was defined as the sum of the following three compartments [15], expressed as percent of the total lung mass: shunt (non-aerated but perfused lung regions, with VNC ≥ − 100 HU and PBV ≥ 1 HU), dead space (aerated, non-perfused regions with VNC < − 100 and PBV < 1), and non-aerated/non-perfused areas (VNC ≥ − 100 HU and PBV < 1). We also computed the gas:blood volume ratio as the ratio of the Vgas to the PBV, normalized so that 1 corresponds to proportionally matched gas and blood distributions (details provided in the Additional file 1).

### Statistical analysis

Data are reported as median (interquartile range), unless otherwise specified. For graphing purposes and to improve readability of plots, histograms are reported as means with standard errors (SEM). We compared data between groups with the Mann–Whitney *U,* *χ*^2^ or Fisher’s exact test, as appropriate. Given the extraordinary circumstances in which the study was conducted, the analysis plan was developed during the conduction of the study. Our co-primary endpoints were the amount of lung aeration (percent of normally aerated lung tissue) and the extent of gas:blood volume mismatch. An a priori sample size calculation was not feasible due to the lack of data on both COVID-19 and quantitative DECT analysis, however, the achieved sample size was higher than or comparable to that of similar imaging studies in COVID-19 [10, 15]. We investigated the correlations between key DECT variables and clinical parameters using Spearman’s rho. In a sensitivity analysis, we used linear regression to model the PaO_2_/FiO_2_ ratio as a function of the extent of aeration-perfusion compartments. All statistical analyses were performed in SPSS Statistics for Windows, Version 25.0 (IBM Corp., Armonk, NY, USA). Significance was assumed at a two-tailed *p*-value < 0.05.

## Results

Overall, 121 critically ill COVID-19 patients were admitted to the ICU during the study period. Thirty-five patients were included in this study (inclusion flow is reported in 

**Fig. 1:**
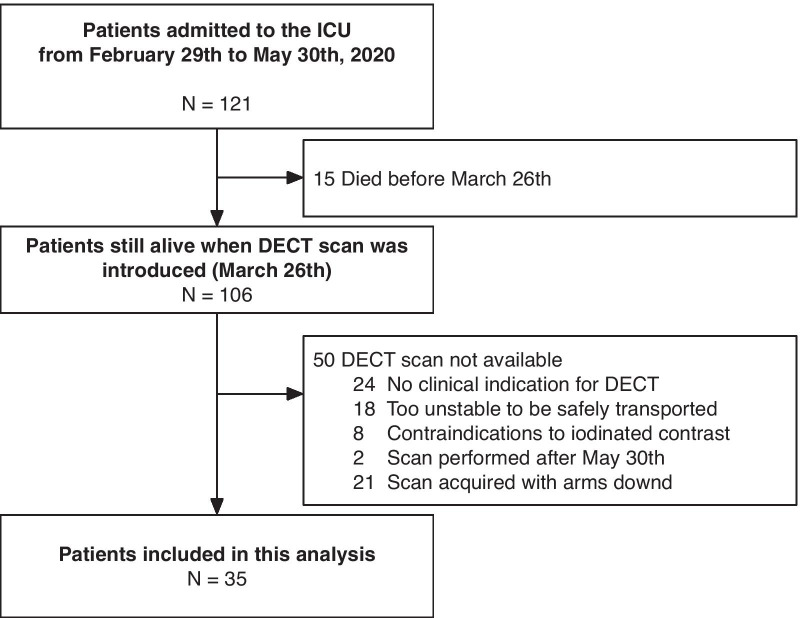
Patient inclusion flow. Date of first admission of a COVID-19 patient: February 29th, 2020; date of introduction of DECT scan in routine practice: March 26th, 2020; last DECT scan included in this analysis: May 30th, 2020

**Fig. 2:**
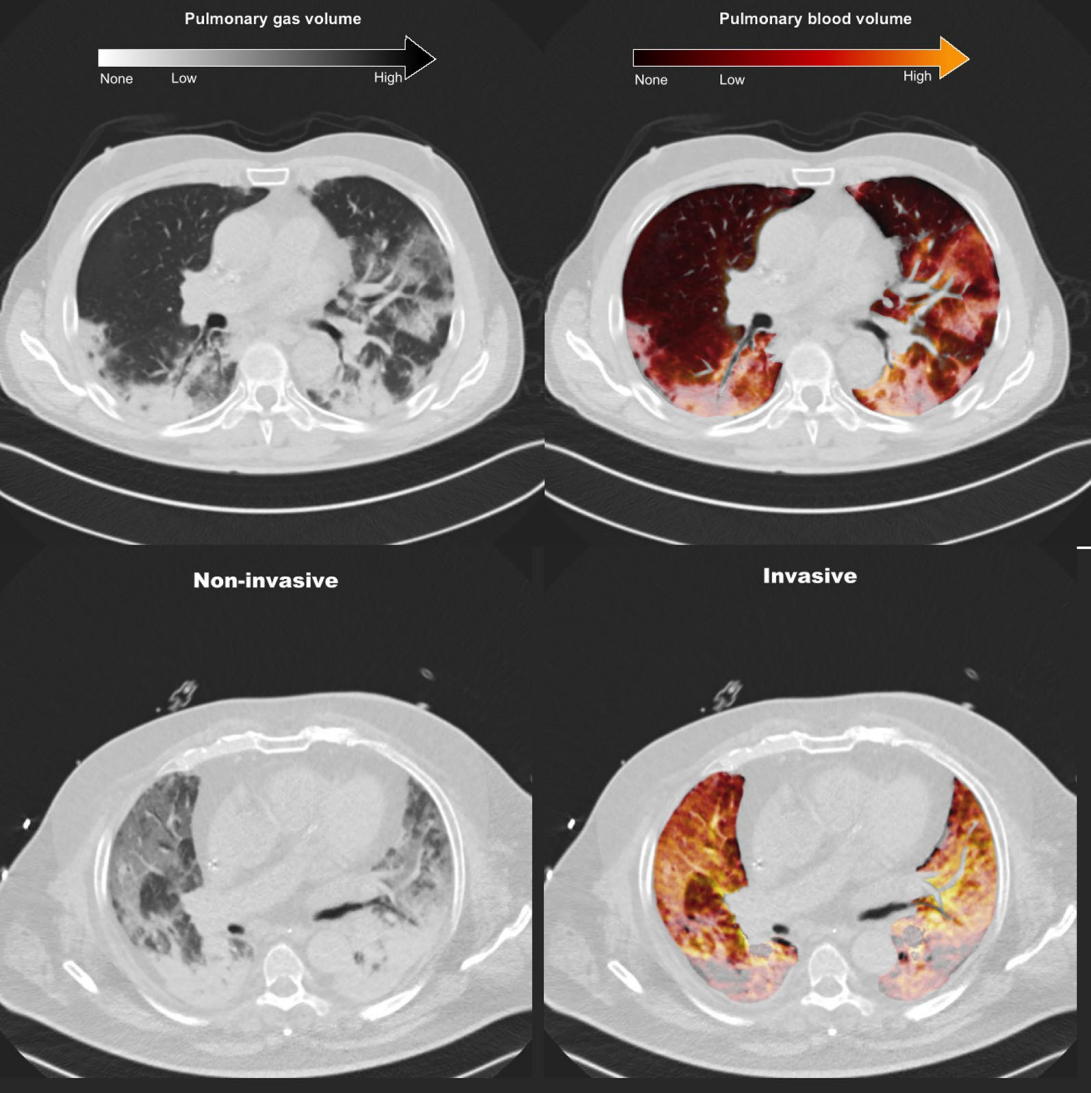
Representative DECT scans of patients receiving non-invasive (top panels) and invasive (bottom panels) respiratory support. The images on the left represent the virtual non-contrast image used for the assessment of aeration, while those on the right represent the pulmonary blood volume map superimposed onto the virtual non-contrast image. The patient in non-invasive ventilation shows areas of ground-glass with both high (yellow) and low (gray) pulmonary blood volume. The patient receiving invasive ventilation shows a more advanced disease characterized by diffuse ground-glass and consolidative lesions, with vast areas of lack of pulmonary blood volume (grey zones) especially in the dorsal dependent regions. *DECT* dual-energy computed tomography

Fig. 1). Figure 2 shows pulmonary gas and blood volume distributions in two representative patients.

### Population description

**Fig. 3:**
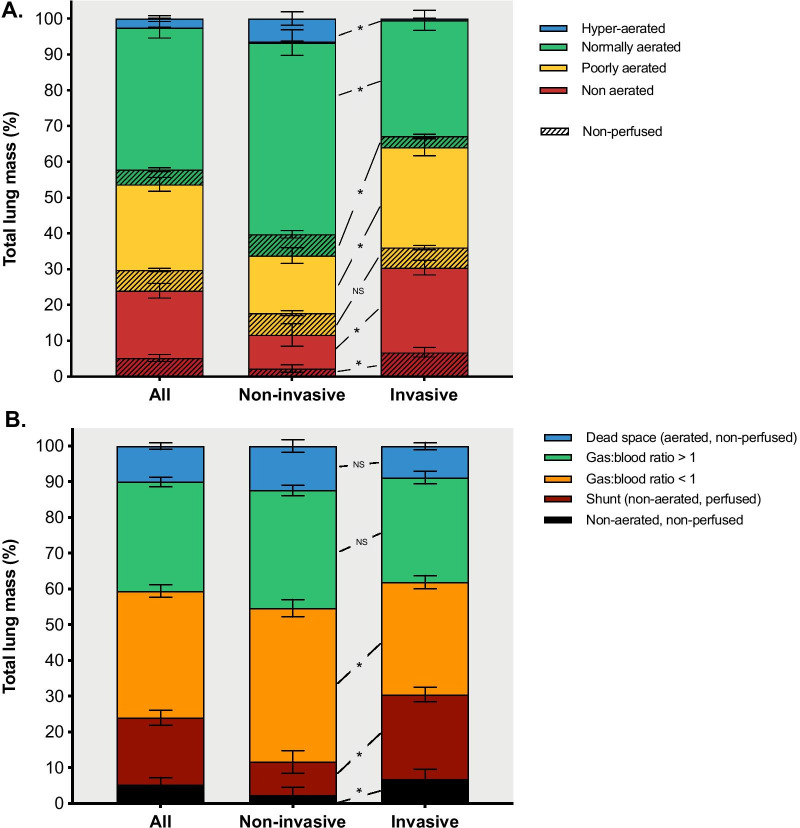
Quantitative DECT analysis in patients receiving non-invasive versus invasive respiratory support. Lung tissue mass, divided into four aeration compartments, is reported as percent of total lung mass (**a**); striped bars represent non-perfused regions. The bottom panel (**b**) illustrates the lung mass divided according to the aeration-perfusion matching compartments. *Significant difference between patients receiving non-invasive and invasive ventilation (*p* < 0.05). *DECT* dual-energy computed tomography. Bars and error bars represent the mean and standard error of the mean, respectively

**Fig. 4:**
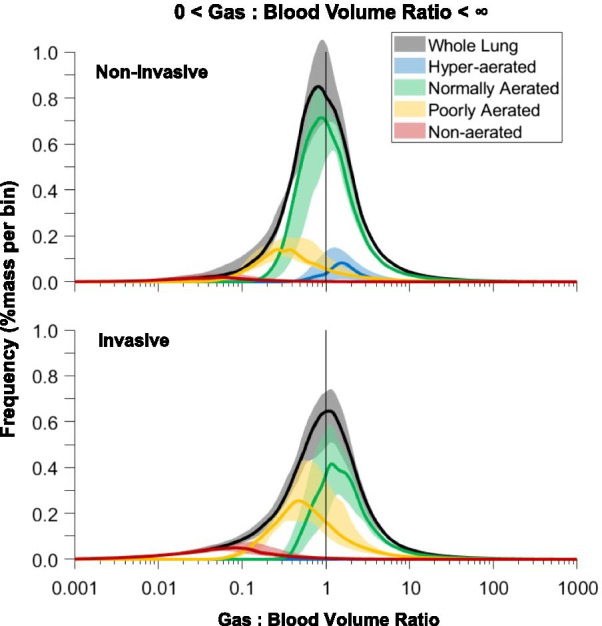
Pulmonary gas:blood volume matching in invasive (upper panel) and non-invasive (lower panel) groups. The curves represent the distribution of voxels according to their gas:blood volume ratio values in the four aeration compartments, where 1 represents voxels with proportionally matched aeration and perfusion

Baseline characteristics of patients and clinical parameters on the day of DECT scan are reported in Table 1. Fourteen patients (40%) died in the ICU, while 21 (60%) were discharged alive to the ward. Compared to patients receiving non-invasive respiratory support, those invasively ventilated had a longer time elapsed from the onset of symptoms, higher levels of D-dimer and inflammatory markers, worse gas exchange impairment (Table 1). The results of quantitative DECT analysis are reported in Table 2 and illustrated in Figs. 3 and 4.

**Table 1:** Characteristics of patients and parameters on the day of the dual-energy computed tomography scan

	All (*N* = 35)	Non-invasive (*N* = 12)	Invasive (*N* = 23)	*p*
Age	59 [55–64]	58 [55–62]	60 [53–67]	0.694
Sex (male), *n* (%)	30	10	20	
Weight (kg)	80 [75–96]	90 [85–96]	80 [73–95]	0.173
Body mass index (kg/m^2^)	28 [25–31]	28 [28–31]	28 [24–32]	0.521
Days since onset of symptoms	19 [16–31]	18 [16–19]	25 [15–35]	0.045*
Days since first confirmed swab	16 [9–22]	12 [9–14]	20 [11–32]	0.026*
Days since hospital admission	13 [7–21]	12 [8–13]	19 [7–31]	0.115
Days since ICU admission	12 [3–21]	12 [8–13]	15 [2–31]	0.161
*Comorbidities*				
Charlson Comorbidity Index	1 [1, 2]	2 [1, 2]	1 [1, 2]	0.878
Hypertension, *n* (%)	14 (40.0)	6 (50.0)	8 (34.8)	0.477
Diabetes, *n* (%)	6 (17.1)	2 (16.7)	4 (17.4)	> 0.999
History of pulmonary embolism, *n* (%)	1 (2.9)	0 (0.0)	1 (4.3)	> 0.999
Chronic obstructive lung disease, *n* (%)	3 (8.6)	2 (16.7)	1 (4.3)	0.266
*Chronic therapy*				
Calcium channel blockers, *n* (%)	2 (5.7)	1 (8.3)	1 (4.3)	> 0.999
Angiotensin II receptor blockers, *n* (%)	4 (11.4)	3 (25.0)	1 (4.3)	> 0.999
Angiotensin-converting enzyme inhibitors, *n* (%)	3 (8.6)	2 (16.7)	1 (4.3)	0.239
Oral anticoagulants, *n* (%)	2 (5.7)	2 (16.7)	0 (0.0)	0.098
Antiplatelet therapy, *n* (%)	5 (14.3)	5 (41.7)	0 (0.0)	0.150
Steroids in the previous month, *n* (%)	3 (8.6)	2 (16.7)	1 (4.3)	0.239
*Drugs received during ICU stay*				
Darunavir/ritonavir, *n* (%)	5 (14.3)	0 (0.0)	5 (21.7)	0.150
Hydroxychloroquine, *n* (%)	24 (68.6)	10 (83.3)	14 (60.9)	0.432
Remdesivir, *n* (%)	1 (2.9)	0 (0.0)	1 (4.3)	> 0.999
Tocilizumab, *n* (%)	12 (34.3)	5 (41.7)	7 (30.4)	0.709
Methylprednisolone, *n* (%)	15 (42.9)	6 (50.0)	9 (39.1)	0.721
*Anticoagulation regimen*				
None, *n* (%)	1 (2.9)	0 (0.0)	1 (4.3)	0.098
Enoxaparin, prophylactic dose, *n* (%)	9 (25.7)	6 (50.0)	3 (13.0)	
Enoxaparin, therapeutic dose, *n* (%)	18 (51.4)	5 (41.7)	13 (56.5)	
Sodium heparin (continuous infusion), *n* (%)	7 (20.0)	1 (8.3)	6 (26.1)	
*Blood analyses*				
Interleukin-6 (ng/L)	45 [8–153]	18 [8–45]	85 [10–739]	0.115
D-dimer (mcg/L)	1497 [990–4126]	1024 [519–2792]	1581 [1174–5358]	0.023*
Ferritin (mcg/L)	921 [603–1888]	733 [477–904]	1399 [888–1929]	0.016*
C-reactive protein (mg/L)	17 [9–89]	10 [5–16]	40 [13–107]	0.017*
*Gas exchange*				
pHa	7.43 [7.40–7.45]	7.44 [7.42–7.47]	7.43 [7.34–7.45]	0.184
PaCO_2_ (mmHg)	43 [39–53]	39 [37–42]	47 [42–55]	0.001*
PaO_2_ (mmHg)	94 [77–125]	108 [93–144]	82 [71–97]	0.023*
PaO_2_/FiO_2_ (mmHg)	179 [117–195]	194 [186–250]	139 [108–188]	0.002*
Bicarbonate (mEq/L)	28 [25–31]	27 [25–28]	29 [25–33]	0.071
*Respiratory parameters*				
PEEP (cmH_2_O)	10 [8–10]	10 [8–10]	10 [8–12]	0.959
FiO_2_ (%)	60 [50–70]	60 [45–63]	60 [50–70]	0.172
Respiratory rate (min^−1^)	19 [16–24]	16 [14–20]	20 [16–25]	0.011*
Tidal volume per predicted body weight (mL/kg)	7.3 [5.5–7.8]	n.a	7.3 [5.5–7.8]	n.a
Driving pressure (cmH_2_O)	16 [12–18]	n.a	16 [12–18]	n.a
Plateau pressure (cmH_2_O)	25 [21–28]	n.a	25 [21–28]	n.a
Compliance (mL/cmH_2_O)	34 [22–43]	n.a	34 [22–43]	n.a
Ventilatory ratio	1.8 [1.4–2.4]	n.a	1.8 [1.4–2.4]	n.a

n.a.: data unavailable in non-intubated patients. ICU: intensive care unit; PEEP: positive end-expiratory pressure; pHa: arterial pH; PaCO2: arterial partial pressure of carbon dioxide; PaO_2_: arterial partial pressure of oxygen; FiO_2_: fraction of inspired oxygen

*Non-invasive versus invasive respiratory support (*p* < 0.05)

**Table 2:** Quantitative lung dual-energy computed tomography parameters

Parameter	All (*N* = 35)	Non-invasive (*N* = 12)	Invasive (*N* = 23)	p
Lung volume (mL)	2794 [2150 to 3690]	3680 [3180 to 4245]	2359 [1934 to 3207]	0.001
Mean attenuation (HU)	− 615 [− 687 to − 454]	− 771 [− 807 to − 649]	− 506 [− 639 to − 406]	< 0.001*
Lung weight^§^ (g)	1039 [878 to 1268]	966 [759 to 1061]	1086 [884 to 1283]	0.045*
Pulmonary gas volume (mL) (Vgas)	1621 [1019 to 2498]	2929 [1873 to 3350]	1140 [726 to 1765]	< 0.001*
Evidence of macroscopic pulmonary embolism, *n* (%)	6 (17.1)	1 (8.0)	5 (21.7)	0.640
Non-perfused tissue (g)	138 [90 to 219]	118 [87 to 201]	180 [102 to 262]	0.327
Dead-space tissue (g)	87 [57 to 162]	87 [71 to 142]	86 [51 to 171]	0.503
Non-aerated/non-perfused lung tissue (g)	33 [12 to 84]	11 [3 to 21]	65 [29 to 105]	0.001*
Shunt tissue (g)	193 [70 to 299]	39 [20 to 128]	253 [180 to 357]	< 0.001*
Tissue with gas:blood volume ratio < 1 (g)	360 [292 to 435]	429 [325 to 464]	355 [291 to 423]	0.195
Tissue with gas:blood volume ratio > 1 (g)	293 [249 to 355]	295 [273 to 328]	293 [248 to 365]	0.932

*HU* hounsfield units

^*^Non-invasive *versus* invasive respiratory support (*p* < 0.05)

^§^Lung weight measured without considering blood vessels; thus, values are lower compared to conventional quantitative computed tomography analysis

### Lung DECT analysis—pulmonary gas volume distribution

The percentage of normally aerated tissue was lower in invasively ventilated patients compared to those receiving non-invasive respiratory support (33% [24–49%] vs. 63% [44–68%], *p* < 0.001, Fig. 3a). Patients receiving invasive compared to non-invasive mechanical ventilation had larger poorly- and non-aerated regions (Fig. 3a), and these increased along the ventral-dorsal (Additional file 1: Fig. S1A) and the apical-caudal (Additional file 1: Fig. S2A) axes. Invasively ventilated patients had lower total lung volume and higher lung weight (Table 2).

### Lung DECT analysis—pulmonary blood volume distribution

Non-perfused regions were distributed in normally-, poorly- and non-aerated regions, as illustrated in Fig. 3a. Non-perfused areas were located mainly in the non-aerated regions in invasively ventilated patients, while those receiving non-invasive respiratory support had larger amounts of non-perfused parenchyma in the normally aerated regions. The incidence of macroscopic pulmonary embolism was numerically higher in invasively versus non-invasively ventilated patients, but this difference was not statistically significant (21.7% vs. 8.0%, *p* = 0.640, Table 2). The amount of non-perfused tissue in patients with (*N* = 6) and without (*N* = 29) radiological evidence of macroscopic pulmonary embolism was 18% [12–23%] and 14% [8–18%], respectively (*p* = 0.272).

### Lung DECT analysis—gas:blood volume matching

The extent of gas:blood volume mismatch was higher in invasively ventilated patients compared to those on non-invasive support (43% [30–49%] vs. 25% [14–28%], *p* = 0.001). Pulmonary blood volume was distributed along a ventral-dorsal and an apical-caudal gradient and its distribution was similar in patients receiving non-invasive and invasive respiratory support (Additional file 1: Fig. S3). Shunt, non-aerated/non-perfused compartments, and areas with gas:blood volume ratio < 1 were higher in invasively ventilated patients compared to those on non-invasive support (Fig. 3b). Non-aerated/non-perfused, shunt, and regions with gas:blood volume ratio < 1 increased along the ventral-dorsal (Additional file 1: Fig. S1B) and the apical-caudal (Additional file 1: Fig. S2B) axes. The poorly aerated compartment (29% [22–34%]) had gas:blood volume ratio both below (21% [16–32%]) and above 1 (5% [4–7%]). The non-aerated compartment (26% [11–36%]) acted as shunt (20% [9–25%]) or was non-perfused (4% [1–8%]). Dead space tissue was 9% [6–14%], distributed both in the normally- (4% [2–6%]) and poorly-aerated (5% [4–7%]) compartments, with negligible amounts in hyperaerated regions. Figure 4 illustrates the distribution of the gas:blood volume ratio in invasively ventilated patients and in those receiving non-invasive support.

### Correlations between clinical and DECT parameters

**Fig. 5:**
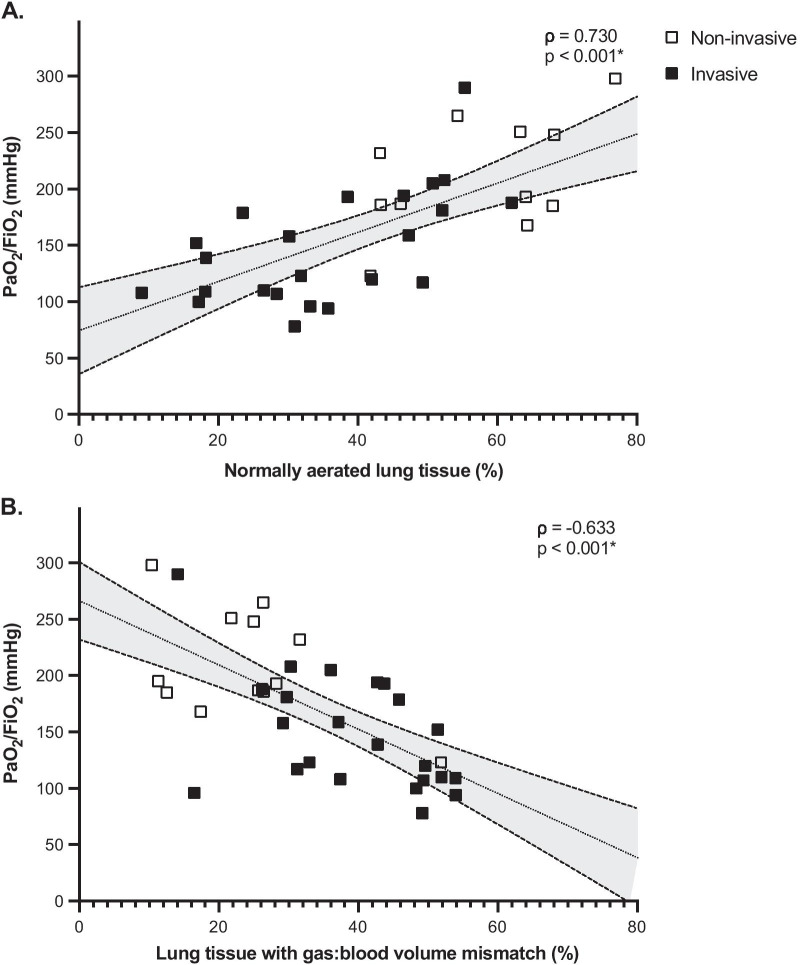
Correlations between PaO_2_/FiO_2_ ratio and lung aeration and gas:blood volume mismatch. Gas:blood volume mismatch is the percent of lung mass accounted for shunt, dead space and non-aerated non-perfused regions. *Significant correlation

Table 3 illustrates the correlations between clinical (D-dimer, C-reactive protein, respiratory system compliance, and blood gas analysis) and DECT parameters. D-dimer levels were correlated with loss of aeration, gas:blood volume mismatch, and extent of non-perfused regions. C-reactive protein levels correlated with loss of aeration and gas:blood volume mismatch. The PaO_2_/FiO_2_ correlated positively with the extent of normally aerated tissue, and inversely with the extent of gas:blood volume mismatch, poorly aerated, and non-aerated compartments (Fig. 5). PaCO_2_ correlated with loss of aeration and gas:blood volume mismatch, while the ventilatory ratio with the total amount of non-perfused lung parenchyma (Table 3). The amount of PBV detected in the non-aerated areas, expressed as percent of the total lung PBV, was not correlated with PEEP levels (*ρ* = − 0.107, *p* = 0.539) nor with the driving pressure (*ρ* = 0.048, *p* = 0.829). In a sensitivity analysis based on a linear regression model using type of respiratory support and aeration-perfusion compartments as covariates, shunt was the only variable independently associated with the PaO_2_/FiO_2_ ratio (Additional file 1: Table S1).

**Table 3:** Correlations between clinical and dual-energy computed tomography parameters

Dual-energy computed tomography parameters	D-dimer C-reactive protein				Respiratory system compliance^§^		PaO_2_/FiO_2_		PaCO_2_		Ventilatory Ratio^§^	
	*ρ*	*p*	*ρ*	*p*	*ρ*	*p*	*ρ*	*p*	*ρ*	*p*	*ρ*	*p*
Normally aerated lung tissue (%)	− 0.403	0.016*	− 0.488	0.003*	0.284	0.189	0.730	< 0.001*	0.483	0.003*	− 0.351	0.101
Poorly aerated lung tissue (%)	0.343	0.044*	0.236	0.172	− 0.144	0.511	− 0.342	0.044*	− 0.221	0.201	0.407	0.054
Non-aerated lung tissue (%)	0.379	0.025*	0.575	< 0.001*	− 0.280	0.196	− 0.637	< 0.001*	− 0.383	0.023*	0.108	0.625
Non-perfused lung tissue (%)	0.418	0.012*	0.061	0.730	− 0.071	0.747	− 0.12	0.491	0.385	0.023*	0.432	0.040*
Shunt lung tissue (%)	0.186	0.285	0.486	0.003*	− 0.105	0.634	− 0.579	< 0.001*	0.261	0.130	− 0.149	0.497
Lung tissue with gas:blood volume ratio < 1 (%)	− 0.418	0.012*	− 0.557	0.001*	0.422	0.045*	0.481	0.003*	− 0.441	0.008*	0.067	0.761
Lung tissue with gas:blood volume ratio > 1 (%)	− 0.285	0.097	− 0.169	0.333	− 0.166	0.449	0.471	0.004*	− 0.227	0.190	− 0.116	0.599
Dead-space lung tissue (%)	0.101	0.565	− 0.351	0.039*	0.165	0.451	0.289	0.092	0.070	0.690	0.205	0.349
Non-aerated, non-perfused lung tissue (%)	0.513	0.002*	0.531	0.001*	− 0.212	0.332	− 0.439	0.008*	0.497	0.002*	0.368	0.084
Lung tissue with gas:blood volume mismatch (%)	0.440	0.008*	0.481	0.003*	− 0.138	0.529	− 0.633	< 0.001*	0.440	0.008*	0.163	0.457

Gas:blood volume mismatch is the percent of lung mass accounted for by shunt, dead-space, and non-aerated, non-perfused regions. FiO_2_: fraction of inspired oxygen; PaO_2_: arterial oxygen partial pressure; PaCO_2_: arterial carbon dioxide partial pressure

^*^Significant correlation, *p* < 0.05

^§^Correlations computed only in intubated patients (*N* = 23)

## Discussion

In the present study, we conducted a quantitative investigation of the lung distribution of pulmonary gas and blood volume in critically ill patients with severe COVID-19 pneumonia. We found that: (1) the percentage of normally aerated tissue was lower, while the extent of gas:blood volume mismatch was higher in invasively compared to non-invasively ventilated patients; (2) the poorly aerated tissue was characterized by either high or low gas:blood volume ratio; (3) the non-aerated lung tissue mass was mostly perfused, with only a small proportion being non-perfused; and (4) the PaO_2_/FiO_2_ ratio correlated with normally aerated lung tissue and with the extent of gas:blood volume mismatch.

This is the first study to evaluate DECT findings quantitatively in severe COVID-19 pneumonia, with different degrees of clinical severity, as reflected by the type of respiratory assistance (non-invasive and invasive respiratory support). In our center, DECT was performed routinely in a high proportion of patients with COVID-19 pneumonia for clinical purposes. Radiological findings were analyzed according to requested level of respiratory support, as well as correlated with key physiological, biological, and clinical parameters. All patients included in the present study were severely hypoxemic and admitted to an ICU, where they received either non-invasive or invasive respiratory support. In other pulmonary diseases, contrast-enhanced DECT has been used to quantify blood volume, percentage of lack of perfusion, and different components of lung aeration and tissue mass [18, 24, 25].

COVID-19 patients receiving invasive mechanical ventilation based on clinical indication, compared to those receiving non-invasive respiratory support, showed reductions in pulmonary and gas volume and increased areas of poorly aerated and non-aerated lung tissue. Furthermore, patients receiving invasive mechanical ventilation had a greater extent of shunt lung tissue and larger non-aerated/non-perfused lung regions but less tissue with a low gas:blood volume ratio compared to those receiving non-invasive respiratory support. Non-aerated regions were mostly perfused, with a small proportion being non-perfused. This is compatible with impairment of hypoxic vasoconstriction [8]. On the other hand, poorly aerated areas, corresponding to ground-glass opacities, could either act as dead space, compatible with hypoxic vasoconstriction and/or microthrombosis, or as regions with low gas:blood volume ratio, thus suggesting partial loss of aeration with relative increase of perfusion due to insufficient or absent hypoxic vasoconstriction. A similar phenomenon was recently hypothesized in a computational model [14]. The contribution of poorly aerated areas in determining gas-exchange impairment could explain why hypoxia in COVID-19 might not be associated with changes in respiratory compliance, as occurs in conventional ARDS [10].

COVID-19 pneumonia is characterized by a progressive deterioration of lung morphology, affecting both aerated and poorly/non-aerated compartments. Increased lung weight is potentially explained by increased edema, cellular infiltration, alveolar consolidation, or a combination thereof. According to a recent study [27], edema might have a less important role in determining increased lung weight as compared to alveolar infiltration and mucinosis, pneumovascular lysis, and fibrosis. This is in line with the findings of a previous study showing that higher PEEP levels in COVID-19 patients did not result in relevant alveolar recruitment [12], as would be expected in case of increased edema and atelectasis formation [28]. Therefore, the limited recruitment and complex effects on aeration-perfusion matching should be taken into account when titrating PEEP in COVID-19 patients.

Aeration and blood volume matching were severely compromised, and areas of shunt, dead space, and non-aeration/non-perfusion coexisted. PaO_2_/FiO_2_ correlated positively with the extent of normally aerated tissue and inversely with the extent of gas:blood volume mismatch, and was independently associated with shunt. We hypothesize that hypoxemia in COVID-19 pneumonia can be explained by shunt due to higher blood volume in non-aerated lung tissue and higher blood volume distribution in normally and poorly aerated tissue, causing an overall reduction in gas:blood volume ratio. On the other hand, a higher percentage of hypoperfusion in non-aerated regions (compatible with hypoxic vasoconstriction, compression of capillaries, and/or micro-thrombosis of peripheral vessels [29–31]) could limit the severity of hypoxia. However, when the amount of nonperfused areas exceeds a certain threshold, this may result in diversion of blood flow towards injured lung areas, contributing to worsening of oxygenation. The distribution of aeration in severe COVID-19 patients was similar to that observed in ARDS, while the distribution of blood volume followed a reverse gravitational pattern—namely, higher blood volume in aerated, ventral lung regions. This distribution of blood volume was thus different from those seen in healthy individuals and non-COVID ARDS [32, 33]. This might be explained by the presence of large non-perfused areas affecting predominantly the middle and dorsal lung regions. Since ROIs were defined based on lung weight, the relative amount of PBV in each ventral-dorsal ROI reflects the amount of blood per unit of lung tissue weight. Moreover, blood vessels were excluded from the segmentation, thus measures of PBV reflect the amount of blood in lung parenchyma, excluding the amount in blood vessels not participating to gas exchange. The pathophysiologic mechanisms of dorsal decrease in PBV could comprise hypoxic vasoconstriction, mechanical compression of capillaries and (micro)thrombosis. Loss of aeration and gas:blood volume mismatch were associated with higher levels of inflammatory markers and increased D-dimer, while non-perfused areas correlated with increased D-dimer and increased PaCO_2_. Patients with less severe disease had larger amounts of lung tissue with a low gas:blood volume ratio but less shunt, possibly confirming the hypotheses on different phenotypes of COVID-19 proposed in the early phase of the pandemic [7–9].

Some limitations of our study should be addressed. Only stable patients with a clinical indication for DECT were included in this study, which may have introduced selection bias and resulted in a small sample size. DECT does not directly detect perfusion, but rather the relative amount of iodine concentration and, consequently, regional blood volume. However, it has been validated as an acceptable surrogate for regional blood flow in ARDS [18, 24]. The key analyses were focused on compartments defined based on the presence or absence of pulmonary blood: regions with blood volume not detected by the DECT correspond to areas where perfusion is negligible or absent. Pronation was done in 7 out of 23 intubated patients and probably did not affect the final results, since DECT was always performed in the supine position and never immediately after prone position.

## Conclusions

In critically ill patients with severe COVID-19 pneumonia, loss of aeration and gas:blood volume mismatch were higher in patients requiring invasive mechanical ventilation compared to those receiving non-invasive respiratory support. The severity of hypoxemia was explained by the extent of loss of aeration and gas:blood volume mismatch.

## Supplementary Information

**Additional file 1**. Additional analyses.

## Data Availability

Dataset available from the corresponding author upon reasonable request.
