# Supplementary material for: Lung distribution of gas and blood volume in critically ill COVID-19 patients: a quantitative dual-energy computed tomography study
Source: Crit Care. 2021 Jun 21;25:214. doi: 10.1186/s13054-021-03610-9 (PMC8215486; doi:10.1186/s13054-021-03610-9)
Supplement: Supplementary file 1 — Additional file 1. Additional analyses. [file 13054_2021_3610_MOESM1_ESM.docx]

**Supplement 1**

**Lung distribution of gas and blood volume in severe COVID-19 pneumonia: a quantitative dual-energy computed tomography study**

Ball L et al.

Table of Contents

Clinical context and rationale for the use of DECT 2

Clinical indications for lung DECT scan 2

Technical details on the quantitative dual-energy computed tomography acquisition and analysis 2

Computational details 4

eFigure 1: Ventral – dorsal analysis 7

eFigure 2: Apical – caudal analysis 8

eFigure 3: Regional distribution of pulmonary blood volume 9

eTable 1: Linear regression of PaO_2_/FiO_2_ as function of gas:blood matching 10

References 11

# Clinical context and rationale for the use of DECT

The first critically ill COVID-19 patient was admitted to our ICU on February 29^th^, 2020, in the context of a national emergency and with a paucity of medical literature concerning the disease. In the following weeks, we observed several cases of both massive and segmental pulmonary embolism, confirmed by contrast-enhanced CT and associated with high morbidity and mortality. Moreover, we noticed a high prevalence of elevated D-dimer levels even in patients without clear evidence of pulmonary embolism, a finding later confirmed by reports from Wuhan, China.(1) For these reasons, we decided to include DECT scan as part of the clinical workup of critically ill patients with COVID-19. The study was performed in a 26-bed ICU, expanded during the peak phase to 39 ICU beds plus 8 high-dependency beds.

# Clinical indications for lung DECT scan

Intensivists involved in clinical management ordered lung DECT scan in the following situations: 1) to guide mechanical ventilation and anticoagulation strategies; 2) in the presence of worsening gas exchange; 3) clinical suspicion of right heart failure and/or a steep increase in D-dimer levels; or 4) persistence of high D-dimer levels despite adequate anticoagulant therapy. We excluded patients with absolute contraindications to iodinated contrast medium exposure, as well as those judged too unstable to be safely transported to the CT facility.

# Technical details on the quantitative dual-energy computed tomography acquisition and analysis

Dual-energy CT images were acquired using a dual-source scanner (Siemens Somatom Flash, Erlangen, Germany) with simultaneous low-energy (80 kVp or 100 kVp) and high-energy (140 kVp) acquisitions. The scan was triggered by monitoring the pulmonary trunk enhancement (sensitivity, 100 HU), with a delay of 7 s to allow iodine distribution into the pulmonary vessels and was performed in the caudocranial direction to minimize streak artifacts in the superior cava and subclavian veins. Whole-lung images were acquired. Images were reconstructed with slice thickness 2 mm, increment 1.5 mm, and kernel D30f with separate reconstructions at 80/100 and 140 kV. Lung segmentation was performed automatically on the high-energy image using a multi-resolution convolutional neural network(2) with exclusion of airways, followed by manual refinement as necessary. Pulmonary vessels were segmented from the low-energy image by another convolutional neural network and excluded from the lung mask.

Spatial distribution of pulmonary blood volume (PBV) within the lung mask was computed using a three-material decomposition algorithm(3). Low- and high-energy CT images were first passed through a 3×3×3 mean filter to reduce image noise. Every lung voxel in dual-energy space was represented by two components: 1) a virtual non-contrast (VNC) component along an axis between points corresponding to pure air (-1000 HU at all energy levels) and pure soft tissue (60 HU at 80 kVp, 57 HU at 100 kVp, and 55 HU at 140 kVp); and 2) a deviation from the air-soft-tissue axis representing enhancement due to contrast material. The iodine dual-energy ratio (i.e., the ratio of low- to high-energy enhancement) was assumed to be 3.01 for 80 kV/Sn140 kV image pairs, and 2.24 for 100 kV/Sn140 kV image pairs. The degree of contrast enhancement (i.e., the magnitude of the difference between the measured intensities and the VNC intensities) was assumed proportional to the amount of regional perfused blood volume (PBV) and correlated well with regional perfusion(4).

Whole-lung analysis of perfusion and aeration was performed on the entire lung mask. Regional analyses were then performed on regions of interest (ROIs) with equal lung weight, partitioned by two planar cuts along either the dorsal-ventral axis or the cranial-caudal axis. Lung weight was assumed proportional to the fraction of gas vs. soft tissue in each voxel, using the density of water as an approximation of soft tissue density(5). In each analysis, ROIs were further subdivided according to voxelwise aeration level, using standard thresholds (-900 HU, -500 HU, and -100 HU to delineate hyper-aerated, normal, poorly aerated, and non-aerated lung)(6).

## Computational details

Every voxel $x$ is represented by low- and high-energy CT intensities $C_{x,L}$ and $C_{x,H}$, respectively. The material decomposition is represented by a system of equations representing measured intensities in terms of the fractional contributions from soft tissue $f_{S}$ (with low- and high-energy intensities $C_{S,L}$ and $C_{S,H}$) and air $f_{A}$ (with low- and high-energy intensities $C_{A,L}$ and $C_{A,H}$), as well as contrast enhancement proportional to iodine concentration $\rho_{I}$ along a fixed slope $R_{I}$ in dual-energy space:

$$\left[ \begin{matrix} C_{x,L} \\ C_{x,H} \\ 1 \end{matrix} \right]=\left[ \begin{matrix} C_{S,L} & C_{A,L} & R_{I} \\ C_{S,H} & C_{A,H} & 1 \\ 1 & 1 & 0 \end{matrix} \right]\left[ \begin{matrix} f_{S} \\ f_{A} \\ \rho_{I} \end{matrix} \right]$$

The slope $R_{I}$ is also known as the dual-energy ratio, which describes the ratio between the increase in low-energy CT intensity and the increase in high-energy CT intensity caused by the presence of the iodinated contrast material. Note that it was assumed that $f_{A}=1-f_{S}$. This system of equations can be further simplified into a two-dimensional system:

$$\left[ \begin{matrix} C_{x,L}-C_{A,L} \\ C_{x,H}-C_{A,H} \end{matrix} \right]=\left[ \begin{matrix} C_{S,L}-C_{A,L} & R_{I} \\ C_{S,H}-C_{A,H} & 1 \end{matrix} \right]\left[ \begin{matrix} f_{S} \\ \rho_{I} \end{matrix} \right]$$

The solution of this two-dimensional system by matrix inversion can be expressed analytically in terms of the measured values for $C_{x,L}$ and $C_{x,H}$ in each voxel:

$$\left[ \begin{matrix} f_{S} \\ \rho_{I} \end{matrix} \right]=\frac{1}{\left( C_{S,L}-C_{A,L} \right)-R_{I}\left( C_{S,H}-C_{A,H} \right)}\left[ \begin{matrix} 1 & -R_{I} \\ -C_{S,H}+C_{A,H} & C_{S,L}-C_{A,L} \end{matrix} \right]\left[ \begin{matrix} C_{x,L}-C_{A,L} \\ C_{x,H}-C_{A,H} \end{matrix} \right]$$

which yields direct expressions for the soft tissue fraction:

$$f_{S}=\frac{\left( C_{x,L}-C_{A,L} \right)-R_{I}\left( C_{x,H}-C_{A,H} \right)}{\left( C_{S,L}-C_{A,L} \right)-R_{I}\left( C_{S,H}-C_{A,H} \right)}$$

and relative iodine concentration:

$$\rho_{I}=\frac{\left( -C_{S,H}+C_{A,H} \right)\left( C_{x,L}-C_{A,L} \right)-\left( C_{S,L}-C_{A,L} \right)\left( C_{x,H}-C_{A,H} \right)}{\left( C_{S,L}-C_{A,L} \right)-R_{I}\left( C_{S,H}-C_{A,H} \right)}$$

After solving for $f_{S}$, virtual noncontrast (VNC) images were formed by projecting the measured intensities onto the two-material subspace of air and soft tissue:

$$\left[ \begin{matrix} \mathrm{VNC}_{L} \\ \mathrm{VNC}_{H} \end{matrix} \right]=\left[ \begin{matrix} C_{S,L} & C_{A,L} \\ C_{S,H} & C_{A,H} \end{matrix} \right]\left[ \begin{matrix} f_{S} \\ 1-f_{S} \end{matrix} \right]$$

The pulmonary blood volume (PBV) was then computed as the magnitude of the difference between the measured intensities and the VNC intensities, coercing nonphysical negative values of $\rho_{I}$ to zero:

$$PBV=\left\{ \begin{matrix} \sqrt{\left( C_{x,L}-\mathrm{VNC}_{L} \right)^{2}+\left( C_{x,H}-\mathrm{VNC}_{H} \right)^{2}} & \rho_{I}\geq0 \\ 0 & \mathrm{otherwise} \end{matrix} \right.$$

A gas-to-blood ratio for each voxel was defined as the ratio of mean-normalized gas fraction ${f_{A}}/{\bar{f_{A}}}$ to mean-normalized pulmonary blood volume $\mathrm{PBV}/{\bar{\mathrm{PBV}}}$:

$$Gas:Blood volume ratio=\frac{f_{A}\cdot\bar{\mathrm{PBV}}}{\mathrm{PBV}\cdot\bar{f_{A}}}$$

This definition ensures that a gas:blood volume ratio of 1 corresponds to proportionately matched aeration and PBV. Note that the distribution of values ${f_{A}\cdot\bar{\mathrm{PBV}}}/{\bar{f_{A}}}$ describes a perfectly aeration-matched blood volume distribution with the same mean as the measured PBV distribution but with every value directly proportional to $f_{A}$, representing the same total blood volume distributed in a manner everywhere proportional to gas fraction. A gas:blood volume ratio of 1 implies that the measured PBV is equal to this aeration-matched blood volume.

# eFigure 1: Ventral – dorsal analysis

**eFigure 1:** Quantitative DECT analysis in three ventral, middle, and dorsal regions of interest with equal lung mass. DECT: dual-energy computed tomography.

# eFigure 2: Apical – caudal analysis

**eFigure 2:** Quantitative DECT analysis in three apical, hilar, and caudal regions of interest with equal lung mass. DECT: dual-energy computed tomography.

# eFigure 3: Regional distribution of pulmonary blood volume

**eFigure 3:** Distribution of pulmonary blood volume in three ventral, middle, dorsal and three apical, hilar, and caudal regions of interest with equal lung mass. The PBV distribution was similar in patients receiving non-invasive (A) versus invasive (B) respiratory support (mixed-effects linear model with a random effect for patient and fixed effects for ROI and type of respiratory support: ROI p<0.001, type of respiratory support p=0.99, interaction p=0.52). PBV: pulmonary blood volume; DECT: dual-energy computed tomography.

# eTable 1: Linear regression of PaO_2_/FiO_2_ as function of gas:blood matching

|  | **Regression coefficient**  (95% confidence interval) | **p-value** | **Variance Inflation Factor** |
| --- | --- | --- | --- |
| Invasive respiratory support (versus non-invasive) | -28.9 (-71.5 – 13.8) | 0.177 | 1.6 |
| Shunt lung tissue (%) | -2.9 (-5.1 – -0.7) | 0.028* | 2.8 |
| Lung tissue with gas:blood volume ratio < 1 (%) | -0.8 (-4.1 – 2.6) | 0.648 | 4.5 |
| Dead space lung tissue (%) | -1.8 (-5.5 – 1.9) | 0.324 | 1.5 |
| Non-aerated/non-perfused lung tissue (%) | -2.9 (-7.6 – 1.7) | 0.209 | 2.8 |

**eTable 1:** Linear regression of the PaO_2_/FiO_2_ ratio in mmHg as function of the percent extension of different lung gas:blood volume ratio compartments. The variables are intrinsically inter-correlated because the compartments are mutually exclusive. However, this resulted in moderate multicollinearity as indicated by the variance inflation factor. Model residuals were normally distributed. Model statistics: Unadjusted R^2^ = 0.465, Adjusted R^2^ = 0.372, Corrected Akaike information criterion = 340, Durbin-Watson statistics = 2.1.

# REFERENCES

1. Zhang L, Yan X, Fan Q, Liu H, Liu X, Liu Z, Zhang Z. D‐dimer levels on admission to predict in‐hospital mortality in patients with Covid‐19. *J Thromb Haemost* 2020;jth.14859.doi:10.1111/jth.14859.

2. Gerard SE, Herrmann J, Kaczka DW, Musch G, Fernandez-Bustamante A, Reinhardt JM. Multi-resolution convolutional neural networks for fully automated segmentation of acutely injured lungs in multiple species. *Med Image Anal* 2020;60:101592.

3. Liu X, Yu L, Primak AN, McCollough CH. Quantitative imaging of element composition and mass fraction using dual-energy CT: three-material decomposition. *Med Phys* 2009;36:1602–1609.

4. Fuld MK, Halaweish AF, Haynes SE, Divekar AA, Guo J, Hoffman EA. Pulmonary perfused blood volume with dual-energy CT as surrogate for pulmonary perfusion assessed with dynamic multidetector CT. *Radiology* 2013;267:747–756.

5. Protti A, Iapichino GE, Milesi M, Melis V, Pugni P, Comini B, Cressoni M, Gattinoni L. Validation of computed tomography for measuring lung weight. *Intensive Care Med Exp* 2014;2:31.

6. Ball L, Brusasco C, Corradi F, Paparo F, Garlaschi A, Herrmann P, Quintel M, Pelosi P. Lung hyperaeration assessment by computed tomography: correction of reconstruction-induced bias. *BMC Anesthesiol* 2016;16:67.
